# The relationship between body image, depression, and breastfeeding attitudes in women with 0–24-month-old infants: a cross-sectional study

**DOI:** 10.1590/1806-9282.20241372

**Published:** 2025-03-31

**Authors:** Resmiye Kaya Odabaş, Rukiye Demir

**Affiliations:** 1 Kocaeli University, Faculty of Health Sciences, Department of Midwifery – Kocaeli, Turkey.; 2 Çanakkale Onsekiz Mart University, Faculty of Health Sciences, Department of Midwifery – Çanakkale, Turkey.

**Keywords:** Body image, Breastfeeding, Diet, Depression

## Abstract

**OBJECTIVE:**

The aim of this study was to determine the relationship between body image, depression, and breastfeeding attitudes in women who had infants aged 0–24 months.

**METHODS:**

The study was cross-sectional and correlational in nature, conducted in Turkey between November 2023 and July 2024, with 282 women who had infants aged 0–24 months (n=282).

**RESULTS:**

Of the women who participated in the study, 46.1% received breastfeeding training during the postpartum period, and 46.2% received breastfeeding training between 7 and 12 months. It was determined that women stopped breastfeeding between months, with the main reason being the perception of insufficient milk (51.2%). The total mean score of the Women's Breastfeeding Attitude Evaluation Scale was found to be 99.53±13.48, the Body Perception Scale was 132.19±28.84, and the Beck Depression Scale was 16.19±10.97. Additionally, if there was a high-level, positive linear relationship between the Breastfeeding Attitude Evaluation Scale and the Body Image Scale total scores (r=0.340, p<0.01), then there was a high-level, negative relationship between the Breastfeeding Attitude Evaluation Scale and the Beck Depression Inventory total score (r=-0.503, p<0.01), and a highly negative relationship was found between the Body Image Scale and the Beck Depression Scale (r=-0.526, p<0.01).

**CONCLUSION:**

It has been concluded that low body image and depression in women who had infants aged 0–24 months negatively affect breastfeeding attitudes. It is recommended to investigate the factors affecting breastfeeding attitudes in postpartum women and to provide support and training that can positively affect breastfeeding attitudes.

## INTRODUCTION

Many factors affect women's breastfeeding attitudes. One of the most important issues that must be taken into consideration in order to maintain breastfeeding is "body perception in breastfeeding women"^1,2^. After giving birth, women may feel distressed by changes in their body, such as stretch marks, loose skin, and altered breast shape. In addition, with these changes brought about by pregnancy, women may experience an increase in their body mass index (BMI) during pregnancy, and this increase is associated with negative body perception. Studies have indicated that body perception plays an important role in women's attitudes toward breastfeeding and that negative body perception negatively affects the breastfeeding process in women^3-6^. However, some studies have also indicated that women who want to lose weight due to negative body perception continue breastfeeding for a longer time to lose weight^7^. Studies have also indicated that negative body perception in breastfeeding women can lead to depressive symptoms such as a constant feeling of sadness, general indifference to the environment, loss of appetite, and decreased self-confidence^8^. Depression, especially during breastfeeding, can affect women's ability to cope with negative body image problems and further exacerbate the depression^6,8,9^. The relationship between postpartum depression in women and many factors has been investigated; however, it has not been associated with breastfeeding attitudes^10^. In this context, it is important to determine the relationship between women's body image and depression during the postpartum period and breastfeeding attitudes. The results obtained will shed light on the care and counseling for the protection of maternal and infant health and future studies.

## METHODS

Online in Turkey between November 2023 and July 2024 through various digital platforms.

### Sampling and participants

The population of the study consisted of women who had infants aged 0–24 months. The calculation of the sample size in the study was based on a study by Güney and Uçar^11^ that examined the effect of body image during pregnancy on breastfeeding attitudes and the postpartum breastfeeding process^11^. In the analysis based on the data of this research, the value was determined to be 246. In order to increase the analysis power of the research, the number of samples was increased, and the study was completed with a total of 282 women (n=282).

The study included women between the ages of 18 and 35 years, with a gestational week of 37 and above, who gave live birth, had a baby aged 0–24 months, could speak and understand Turkish, used a smartphone, had internet access, and volunteered to participate in the study. Women with psychiatric disorders, contraindications to breastfeeding (such as flat nipples), and congenital anomalies in their infants were excluded from the study.

### Data collection tools

It was collected online from various digital platforms (Instagram, Facebook, Telegram, and WhatsApp) using the snowball method. The link to the research form was sent via digital platforms to women who met the inclusion criteria. In the introduction section of the form, there was an explanation about the purpose of the research in the form of informed consent and a box where women could give their consent for the research. Women were able to begin answering the questions after reading the consent form on the first page and selecting the confirmation option to indicate their agreement to participate in the study. Each woman who consented to the study and filled out the form was asked to send the survey link to women she knew and who met the sample criteria. Researchers’ bias was prevented by using the snowball sampling method during the data collection process to impartially select the sample group and collect the data independently. The data of the study were collected with "Personal Information Form, Body Image Scale (BIS), Breastfeeding Attitudes of The Evaluation Scale (BAES), and Beck Depression Scale (BDS)."

Personal Information Form: The Personal Information Form was created by the researchers in line with the literature^3,5,6^.

Breastfeeding Attitude Evaluation Scale: The BAES was developed by Arslan Özkan^12^ to evaluate the breastfeeding attitudes of mothers who gave birth. The five-point Likert scale consists of a total of 46 questions. The total score of the scale was 184, and the higher the score received from the scale, the more positive the breastfeeding attitude was. The Cronbach's alpha value of the scale was found to be 0.63, and in this study, it was 0.69^12^.

Body Image Scale: The BIS, which measures how satisfied people are with their bodies and body functions, was developed by Secord and Jourard^13^. The BIS, which was validated in Turkey by Hovardaoğlu and Özdemir^14^, is a 5-point Likert-type measurement tool consisting of 40 items. The lowest total score that can be obtained from the scale is 40 and the highest is 200^14^. Cronbach's alpha value of the scale was found to be 0.95 and 0.96 in this study.

Beck Depression Scale: The BDS was developed by Beck et al.^15^ to objectively determine the degree of depression of the person, and the validity and reliability study in Turkey was conducted by Hisli et al.^15,16^. The total score of the BDS ranges between 0 and 63, and the higher the total score obtained from the scale, the higher the level of depression. The Cronbach alpha coefficient of the scale is 0.73, and 0.94 was found in this study.

### Statistical analysis

Data from the research Statistical Package for the Social Sciences were analyzed using Version 22. As descriptive statistics, mean value, standard deviation, median, minimum, maximum values, and 95% confidence interval were calculated for continuous variables. For categorical variables, the number and percentage values were calculated. A multiple linear regression analysis was performed using the enter method to evaluate the relationship between breastfeeding attitude, body image, and depression. In the analysis of the data, a significance level was set as p<0.05.

### Ethical considerations

Written permission was obtained from Çanakkale Onsekiz Mart University Graduate Education Institute Ethics Committee (Protocol No: 2023-YÖNP-0772 date: 30/10/2023) for the implementation of the research. The women included in the study were informed about the research, and their consent was obtained.

## RESULTS

The demographic characteristics of the women are presented in Table 1. It was found that 46.2% of the women who stopped breastfeeding did so between the 7th and 12th months, with the main reason being the perception of insufficient milk (51.2%) (Table 1).

**Table 1:** Sociodemographic and obstetric characteristics of women (n=282).

Features		n	%
Education level			
	Primary and secondary school	36	12.8
	High school	67	23.8
	University	179	63.4
Working status			
	Working	147	52.1
	Not working	135	47.9
Birth type			
	Vaginal birth	140	49.6
	Cesarean birth	142	50.4
Current breastfeeding status			
	Breastfeeding	204	72.3
	Nonbreastfeeding	78	27.7
Time to stop breastfeeding (months) (n=78)*			
	First 6 months	23	29.4
	7–12	36	46.2
	13–18	12	15.5
	19 and over	7	8.9
Dieting status			
	Yes	95	33.7
	No	187	66.3
The BMI category			
	Normal weight	149	52.9
	Overweight	104	36.9
	degree I obesity	29	10.3
	Mean age±SD** (years) (min–max)	28.97±3.95 (18–35)	
	Postpartum period mean±SD** (months) (min–max)	11.43±6.34 (1–24)	
	BMI±SD** (kg/m^2^) (min–max)	25.02±3.50 (18.7–35.0)	

* Multiple answers provided,

** Standard deviation.

BMI: body mass index; SD: standard deviation.

In this study, while a high-level, positive linear relationship was found between the total scores of ETDS and VAS (r=0.340, p<0.01), a high-level, negative relationship was found between ETDS and total score of BDI (r=-0.503, p<0.01). In addition, a high-level, negative correlation was found between VAS and BDI (r=-0.526, p<0.01) (Table 2).

**Table 2:** Correlation coefficients between Breastfeeding Attitudes of The Evaluation Scale, Body Image Scale, and Beck Depression Scale correlation coefficients.

Variable	BAES	BIS	BDS
BAES	1	0.340*	-0.503*
BIS		1	-0.526*
BDS			1

n=282,

* p<0.01. BAES: Breastfeeding Attitudes of The Evaluation Scale. BDS: Beck Depression Scale. BIS: Body Image Scale.

In this study, a linear regression analysis was performed using the enter method to explain the relationship between women's breastfeeding attitudes, body perception, and depression, and the model was found to be statistically significant (p<0.001; F=46.040). In other words, body perception and depression had a significant effect on women's breastfeeding attitude (p=0.049 and p≤0.001) (Table 3).

**Table 3:** Women's Breastfeeding Attitudes of The Evaluation Scale total mean scores and Body Image Scale and Beck Depression Scale scores by linear regression analysis.

		B (95%CI)*	Beta	t	p	Zero-order	Partial
Enter method							
	Constant	100.485 (91.433–109.538)	–	21.851	<0.01	-	-
	BIS	0.557 (0.00–0.113)	0.121	1.977	0.049	0.345	0.118
	BDS	-0.520 (-0.669 to −0.372)	-0.423	-6.920	<0.01	-0.487	-0.383

B: Unstandardized coefficient; Beta: standardized coefficient; CI: confidence interval; BIS: Body Image Scale; BDS: Beck Depression Scale.

* Confidence interval, F=46.049; p=0.000; Adj. R^2^=0.243; standard error=11.734.

## DISCUSSION

In this study, which was conducted to determine the relationship between body perception, depression, and breastfeeding attitude in women who had infants aged 0–24 months, we determined that as women's positive body perceptions increased, their breastfeeding attitudes also increased. Similarly, in a study examining the effect of body image on a woman's postpartum breastfeeding attitude and breastfeeding process, it was found that as the average score of perceived body image during pregnancy increased, attitudes toward breastfeeding in the postpartum period increased positively^17^. Brown et al. determined that women with body image concerns during pregnancy were more likely to use ready-made formulas and keep the breastfeeding period shorter in the postpartum period^18^. In addition, evaluating women's body perception and how they are affected by this perception in the postpartum period, as well as understanding the woman's response to her body perception and the influence of others’ attitudes, is important for developing positive breastfeeding behavior and maintaining breastfeeding.

It has been reported that successful breastfeeding depends not only on physiological factors but also on the psychological state of the woman, and that mental disorders such as depression, anxiety, and stress negatively affect breastfeeding functions^19^. In our study, we observed that as the postpartum depression status of the women participating in the study increased, their breastfeeding attitudes decreased. Studies have shown that women with postpartum depression interact less with their infants, the risk of early discontinuation of exclusive breastfeeding increases, and that there is a positive relationship with the baby's malnutrition and negative breastfeeding attitudes^19-22^. Mothers who are prone to depression have a harder time coping with possible problems, may lose self-confidence more quickly, and may experience stress. This stress experienced by the mother may lead to anxiety regarding the baby care and may prevent her from successfully breastfeeding, which can negatively affect infant feeding attitudes^23^.

It was determined that there was a negative relationship between the positive body perceptions of the women participating in the study and their depression status. This result of our study is similar to the literature, and when the literature is examined, mothers who gain more weight in the postpartum period complain significantly more about their general body image and have more depressive symptoms^24^. In addition, Ayran and Karahan Yılmaz^20^ conducted a study to determine the body image and perceived stress levels of breastfeeding mothers with 0–6-month-old infants, in which they found that as the positive body perception of mothers increases, their stress levels also decrease^20^. Therefore, considering that negative body perception triggers depression in women and this negatively affects women's breastfeeding attitudes, it is extremely necessary to develop positive body perception in breastfeeding women.

## CONCLUSION

The study concluded that as women's positive body perceptions increase, their breastfeeding attitudes increase, their depression states decrease, and postpartum depression negatively affects women's breastfeeding status. Considering the benefits of breast milk and breastfeeding on mother and baby health, it is recommended to support the mother in breastfeeding, investigate the negative factors affecting the breastfeeding process, consider postpartum body image and depression, and conduct further research in this field.
